# OP41 Pharmaceutical Technologies Conditionally Approved By The National Institute For Health And Care Excellence: A Critical Analysis

**DOI:** 10.1017/S026646232400103X

**Published:** 2025-01-07

**Authors:** Yankier Pijeira Perez, Dyfrig Hughes

## Abstract

**Introduction:**

There are concerns with the National Institute for Health and Care Excellence (NICE) conditional approval process with respect to the quality and reliability of the methods of evidence generation, analysis, and reporting; and on the increasing focus on OwR (only with research) in preference to OiR (only in research). Our study critically appraises the methods, quality, and risk of bias of the evidence generated in response to NICE conditionally approved technologies.

**Methods:**

Our study reviewed pharmaceutical technologies appraised by NICE: technology appraisals approved between March 2000 and September 2022 and highly specialized technologies approved up to October 2023. From those, we identified appraisals with OiR and OwR conditional recommendations, and summarized the evidence requested by NICE as part of conditional approval. Then, evidence resubmitted to NICE for reappraisal was analyzed for its compliance with NICE’s initial recommendations for further research and assessed using the Cochrane Collaboration’s tools for risk of bias in randomized trials and the ROBINS-I tool for the non-randomized evidence.

**Results:**

NICE made 54 conditional recommendations relating to technology appraisals (TAs) (13 OiR and 41 OwR) and five highly specialized technologies (HSTs). From those, 16 TAs presented additional evidence for reappraisal [nine OiR and seven OwR] and three HSTs [three OwR]. Two of the nine reappraised TAs with OiR recommendation and four of the seven OwR fully complied with NICE’s request for further evidence. All three HSTs complied in full. However, the majority of reappraised TAs and HSTs included evidence that was deemed to be at serious, high, moderate, or unclear risk of bias.

**Conclusions:**

The quality of evidence presented to NICE following conditional approval varies considerably. There is often widespread noncompliance with requests for further research when conditionally approving a pharmaceutical technology. Complying with NICE’s requests, however, does not necessarily guarantee high-quality evidence when the technology is reappraised. Evidence generated in response to NICE conditional approval recommendations should be subject to quality standards.

